# Designing the optimal JJ-TRIALS study: EPIS as a theoretical framework for selection and timing of implementation interventions

**DOI:** 10.1186/1940-0640-10-S1-A29

**Published:** 2015-02-20

**Authors:** Danica Knight, Steven Belenko, Angela Robertson, Tisha Wiley, Gail Wasserman, Carl Leukefeld, Ralph DiClemente, Gene Brody, Michael Dennis, Christy Scott

**Affiliations:** 1 Institute of Behavioral Research, Texas Christian University, Fort Worth, TX, 76129, USA; 2 Department of Criminal Justice, Temple University, Philadelphia, PA, 19122, USA; 3 Social Science Research Center, Mississippi State University, Starkville, MS, 39762, USA; 4 Services Research Branch, National Institute on Drug Abuse, Rockville, MD, 20852, USA; 5 Columbia University, New York, NY, 10027, USA; 6 College of Medicine, University of Kentucky, Lexington, KY, 40506, USA; 7 Center for AIDS Research, Emory University, Atlanta, GA, 30322, USA; 8 Center for Family Research, University of Georgia, Athens, GA, 30602, USA; 9 GAIN Coordinating Center, Chestnut Health Systems, Normal, IL, 61761, USA; 10 Lighthouse Institute, Chestnut Health Systems, Chicago, IL, 60610, USA

## 

Juvenile Justice-Translating Research Interventions for Adolescents in the Legal System (JJ-TRIALS; a cooperative implementation science initiative launched by NIDA in July 2013) seeks to reduce unmet substance use disorder (SUD) needs by assisting JJ agencies in their efforts to implement best practices and improve SUD service utilization along a behavioral health cascade (screening, assessment, referral, and treatment). Such efforts require systems-level change; thus, the JJ-TRIALS study targets JJ agencies and the behavioral health partners to which juveniles are referred (i.e., providers of SUD services).

Aaron’s implementation science framework [1] provides the foundation for study design and measurement. EPIS conceptualizes change processes as involving four phases: Exploration, Preparation, Implementation, and Sustainment (EPIS). The development of the implementation intervention components, the timing of components, and the measurement of process improvement activities are guided by Aarons’ EPIS model. For instance, data-driven decisionmaking (DDDM) templates and tools will provide basic support for goal selection during the Exploration phase. EPIS also has implications for measurement of process improvement activities. While the four phases are presented linearly, improvement activities may be somewhat recursive, with sites revisiting earlier phases when modifications in their action plans are needed (e.g., reworking Preparation plans after initial Implementation).

Using a clustered randomized design, JJ-TRIALS will compare two implementation interventions: a Core Intervention, involving DDDM strategies to promote change across the EPIS phases, versus an Enhanced Intervention, providing support for DDDM through facilitation and inter-agency change teams. A total of 36 sites representing 7 states and the District of Columbia will be randomized to Core (n = 18) or Enhanced (n = 18) and to one of three start times. Primary research questions address whether DDDM strategies and facilitation of DDDM tools/implementation teams improve: a) the provision and quality of services along a behavioral health cascade (screening, assessment, referral, and treatment of youth with SUD); and b) attitudes toward/use of best practices among staff working with justice-involved youth. Exploratory research questions focus on aspects of the implementation process, inter-organizational collaboration, costs associated with each study arm, and youth outcomes.
